# Distress symptoms and alcohol consumption: anxiety differentially mediates drinking across gender

**DOI:** 10.3389/fpsyg.2023.1191286

**Published:** 2023-07-31

**Authors:** Oscar V. Torres, Justin C. Estep, Mary Gwin, Nicholas P. Aramovich, Giovanni Thomas, Lan Villalta

**Affiliations:** ^1^Department of Behavioral Sciences, San Diego Mesa College, San Diego, CA, United States; ^2^Department of Social Sciences, San Diego Mesa College, San Diego, CA, United States; ^3^Department of Behavioral Sciences, San Diego Miramar College, San Diego, CA, United States

**Keywords:** alcohol, drinking, anxiety, depression, gender differences, sex differences

## Abstract

**Introduction:**

The consumption of alcohol remains a significant health concern and represents a prevalent form of substance use worldwide. Previous research has identified sex differences in the consumption of alcohol. This study explores the relationship between drinking and the presence of distress symptoms across gender. Based on previous research, it was hypothesized that presence of distress symptoms, defined as increases in anxiety and depression, would be prominent features associated with alcohol consumption among women compared to men.

**Methods:**

A sample of undergraduate students (*N* = 448) participated in an online-based questionnaire (71% female; *M* age = 22.1; 42.9% Hispanic/Latino). The questionnaire contained assessments related to demographic information and alcohol consumption over the past 30 days. Anxiety and depression symptoms were assessed using the Hospital Anxiety and Depression Scale.

**Results:**

Gender differences were observed with men consuming more alcoholic drinks than women. However, women who consumed alcohol reported having more distress symptoms relative to their male counterparts. A logistic regression revealed that this gender difference was moderated by anxiety, such that greater anxiety scores associates with whether women consumed alcohol. However, an ordinary least squares regression revealed that for men, anxiety scores significantly related to the amount of drinks consumed. Gender differences were not detected in relation to alcohol consumption and depression.

**Conclusion:**

Our findings contribute to the literature by indicating that the mere presence of distress symptoms reveals distinctive gender-specific differences in relation to alcohol consumption in a non-clinical population. Identifying the distinct associations linked with alcohol use for men and women can aid in reducing drinking disparities among young adults.

## 1. Introduction

In the United States, excessive drinking is a major health concern characterized by increased alcohol-related mortality rates over the past 5 years (Esser et al., 2022; White et al., 2022). In addition, increasing alcohol-associated hospitalizations (White et al., 2018) have led to economic burdens such as growing medical healthcare costs (Peterson et al., 2021; Barbosa et al., 2023). Despite this public health crisis, 55.9% of adults living in the United States consume alcohol at least once a month (Substance Abuse and Mental Health Services Administration, 2019), with an estimated 40% of male and 30% of female college students engaging in binge drinking (Krieger et al., 2018).

A review of the literature reveals many gender differences leading to sex disparities associated with alcohol use (Becker et al., 2017; White, 2020). For example, men consume greater amounts of alcohol compared to women (Erol and Karpyak, 2015). However, data from the United States indicates that these gender gaps are narrowing given the increased rates of binge drinking and occurrence of alcohol misuse among women (White et al., 2015; McKetta and Keyes, 2019). These trends are alarming given that women are at higher risk of developing alcohol-induced physical illnesses including cardiovascular disease (Agabio et al., 2016), liver disease (Yoon et al., 2020), and cognitive deficiencies following chronic drinking (Nixon et al., 2014). Moreover, women may develop sex-specific health damages following hazardous alcohol consumption including hormonal deregulation, pregnancy-related complications, and early menopausal symptoms (Erol and Karpyak, 2015). Women who drink may also encounter a higher prevalence of alcohol-associated traumas such as intimate partner violence (Dardis et al., 2021). Thus, distinct medical and social consequences occur in response to alcohol consumption across gender, and providing research-based information regarding the use of alcohol may guide young adults in deciding when and how much to drink.

Among the many factors facilitating alcohol use, distress symptoms uniquely contribute to gender differences in drinking (Verplaetse et al., 2018; Peltier et al., 2019). For example, women who experience stressful life events have a greater likelihood of developing alcohol use disorder compared to men (Verplaetse et al., 2018). Among college students, women report drinking to cope with negative emotions (Buckner et al., 2023) and experience more distress symptoms in conjunction with drinking (Pedrelli et al., 2016; Perrotte et al., 2018). Similarly, women with binge drinking patterns display higher levels of anxiety (Adan et al., 2016) and those with frequent episodic drinking report less tolerance for distress (Pedrelli et al., 2018). However, there are inconsistencies when considering how components of internalizing distress relate to alcohol use for each gender. For instance, depression symptoms are noted to significantly correlate with drinking behavior for men (Pedersen, 2013). In contrast, other findings suggest that there are no gender differences between drinking and distress symptoms among college students (Nourse et al., 2017).

To further elucidate the relationship between internalizing distress, alcohol consumption, and gender, the present study explored whether the presence of anxiety or depression symptoms distinctly relate with drinking for either gender. A questionnaire was developed to examine anxiety, depression, and drinking behavior in college students. A logistic regression was then used to explore whether anxiety or depression symptoms moderated the relationship between alcohol use across gender. Lastly, ordinary least squares (OLS) regressions were performed to evaluate the relation between gender, anxiety, depression, and the total number of drinks consumed by survey respondents during the past 30 days. Herein, it was hypothesized that women who reported consuming alcohol would report greater anxiety and depressive symptoms relative to their male counterparts.

## 2. Materials and methods

### 2.1. Sample and procedure

Study participants consisted of 448 undergraduate students. Recruitment was conducted at San Diego City College, San Diego Mesa College, and San Diego Miramar College. As an eligibility criterion, participants must have been at least 18 years of age at the time of data collection. Informed consent forms were first distributed which contained language on the voluntary nature of participation based on the NIH Office of Extramural Research directives for the protection of human subjects. Subsequently, flyers containing QR codes linking mobile devices to an online questionnaire were provided. All responses were confidential and anonymous. Institutional review board approval was obtained prior to all data collection (IRB# 2019010).

### 2.2. Measurements

Sociodemographic characteristics included participants’ self-identified gender, age, ethnicity, household income, marital status, employment status, education level, and veteran status. Ethnicity was categorized as either Caucasian, Hispanic/Latino, Asian, African American, or Pacific Islander. Annual household income was categorized as lower income (less than 25 k USD), low to middle income (26 k to 49 k USD), median income (50 k to 74 k USD), middle to upper income (75 k to 100 k USD), and upper income (more than 100 k USD). Based on the distribution of academic experience, educational level was classified as having no college experience, some college experience, or already attained a professional degree. Participants were also asked if they had “Any previous personal psychiatric history” and “Any previous family psychiatric history.”

Alcohol consumption was measured by asking participants if they had any alcohol to drink in the past 30 days. For data analysis, individuals who self-reported consuming alcohol were categorized as “drinkers.” The total number of alcoholic drinks consumed by participants was measured by asking survey respondents to indicate their cumulative number of drinks consumed over the past 30 days. For data analysis, participants who responded “None” or “0” were categorized as “non-drinkers.” Participants were also asked, “Do any of your close friends drink alcohol?” to evaluate the association between respondents who consume alcohol and peer drinkers. These measurements and operational definitions were constructed based on a previous report examining alcohol consumption in a college student sample (Martin et al., 2021).

The presence of internalizing distress symptoms was assessed using the Hospital Anxiety and Depression Scale (HADS), originally described by Zigmond and Snaith (1983). The HADS is a 14-item self-assessing instrument for perceived symptoms of anxiety (HADS-A) and depression (HADS-D). Each subscale contains seven items on a 4-point Likert scale scoring from “0 = not at all” to “3 = nearly all the time.” Sample statements on the HADS-A include “Worrying thoughts go through my mind” and “I get sudden feelings of panic.” Sample statements on the HADS-D include, “I feel as if I am slowed down” and “I have lost interest in my appearance” (Zigmond and Snaith, 1983). Each subscale ranges from 0 to a maximum of 21, with higher scores indicating a worsening of symptoms. Scores ranging from 0 to 7 indicate the non-existence of problematic conditions. Scores ranging from 8 to 10 indicate only mild symptomatology. Scores of 11or higher reflect potential clinical caseness and merit further evaluation for psychiatry disorders. In the present study, Cronbach’s α based on the seven items on the HADS-A was 0.82, and Cronbach’s α based on the seven items on the HADS-D was 0.72.

### 2.3. Data analysis

Sociodemographic data was examined using descriptive statistics. Mean differences between alcohol use, educational level, and psychiatric history were analyzed using *t-*tests or *F*-tests as appropriate. Zero-order correlations were then used to examine associations between variables of interest. To explore gender differences in relation to drinking and generalized internalizing distress, the average number of drinks and combined HADS-D and HADS-A scores were analyzed using *t*-tests. One-way ANOVAs were then conducted to examine potential gender differences in anxiety and depression scores for drinkers and non-drinkers.

To examine whether gender, depression, or anxiety symptoms associated with alcohol user status (e.g., either consuming alcohol or not in the past 30 days), a logistic regression analysis was performed. Entered in Step 1 were participants’ gender, HADS-D scores, and HADS-A scores. Entered in Step 2 were interactions between gender, HADS-D and HADS-A scores to determine if these components improved the overall model.

To explore whether gender, depression or anxiety symptoms relate to the number of drinks consumed (e.g., total reported drinks in the past 30 days) specifically for drinkers, we conducted an OLS regression. Entered in Step 1 were participants’ gender, HADS-D and HADS-A scores. Entered in Step 2, interaction effects between gender, HADS-D and HADS-A scores were included to evaluate if these components improved the overall model. These statistical approaches were based on a previous report (Pedrelli et al., 2011).

To explore whether clinical caseness (e.g., high scores reflecting possible clinical conditions) relate to the number of drinks consumed between men and women, a similar OLS regression was performed. However, each participant’s HADS-D and HADS-A values were dichotomized by lower scores ranging from 0 to 10, and by higher scores ranging from 11 to 21. This approach resulted in clinical and non-clinical cut-off thresholds that determine the existence of potential anxiety or depression problems within our sample. Entered in Step 1 were participants’ gender, clinical HADS-D and clinical HADS-A scores specifically for drinkers. Entered in Step 2 were interaction effects between these variables. All data were analyzed using IBM SPSS Statistics for Windows, version 20.0 (IBM Corp., Armonk, N.Y., United States). All analyzes were considered statistically significant when *p* ≤ 0.05.

## 3. Results

### 3.1. Sociodemographic and alcohol-use related characteristics

Table 1 shows demographic data for non-drinkers and drinks separated by gender. The majority of participants consisted of women (71%) and most common ethnicity was Hispanic/Latino (42.9%). On average participants were 22 years of age, an important characteristic given that the legal drinking age in the United States is 21. In relation to drinking, 60% of the total sample reported consuming alcohol at least once during the past 30 days. Participants with more college experience consumed more drinks (*M* = 6.8, SD = 12.4) compared to those with less college experience (*M* = 2.4, SD = 5.4), or those with professional degrees (*M* = 3.6, SD = 6.0), *F*(2, 447) = 4.16; *p* < 0.05. There were no significant differences between estimated household income and alcohol use *F*(4, 259) = 0.75; *p* = 0.55. Veterans reported drinking more (*M* = 20.1, SD = 27) compared to non-veterans (*M* = 9.2, SD = 11.2), *t*(265) = 3.81, *p* < 0.01. Zero-order correlations revealed moderate relations between alcohol use and sociodemographic characteristics (Table 2).

**Table 1:** Basic demographic data separated by drinking status for men and women.

Variable	Male non-drinkers (*n* = 58)	Male drinkers (*n* = 71)	Female non-drinkers (*n* = 122)	Female drinkers (*n* = 197)	Total sample (*N* = 448)
*Age	22.1 (±0.75)	23.7 (±0.59)	21.6 (±0.56)	21.9 (±0.30)	22.1 (±0.24)
Ethnicity
Caucasian	31.0%	35.2%	18.0%	35.0%	29.9%
Hispanic/Latino	25.9%	36.6%	47.5%	47.2%	42.9%
Asian	24.1%	16.9%	15.6%	10.7%	14.7%
African-American	13.8%	7.0%	13.1%	5.1%	8.7%
Pacific Islander	5.2%	4.2%	5.7%	2.0%	3.8%
Annual household income
Low	36.4%	39.5%	39.7%	43.3%	40.8%
Low-middle	30.9%	39.4%	27.3%	27.3%	29.7%
Middle	12.7%	2.8%	16.5%	9.8%	10.9%
Middle-upper	9.1%	5.6%	10.7%	7.2%	8.2%
Upper	10.9%	12.7%	5.8%	12.4%	10.4%
Bachelor’s degree or higher	5.2%	1.4%	2.5%	2.5%	2.7%
Currently employed	50.0%	53.5%	54.1%	72.6%	61.6%
Married or cohabiting	12.1%	8.4%	10.6%	20.3%	14.8%
Veteran	10.3%	21.1%	2.5%	4.6%	7.4%
Familial psych. history	29.3%	25.4%	23.0%	36.0%	29.9%
Personal psych. history	19.0%	31.0%	23.0%	25.9%	25.0%
Close friend who drinks	70.7%	94.4%	74.6%	98.0%	87.5%

Percentages are based on total number within each respective category. *Indicates average age with standard error of the mean (SEM) in parenthesis. For annual household income, low income: < USD 25 k; low-middle income: USD 25 k-50 k; middle income: USD 50 k-75 k; middle-upper income: USD 75 k-100 k; upper income: > USD 100 k.

**Table 2:** Zero-order correlations between descriptive data and alcohol–related characteristics.

Correlation matrix of alcohol-related variables												
	1	2	3	4	5	6	7	8	9	10	11	12
1. Gender	−											
2. Age	0.11*	−										
3. Employment	−0.13**	−0.07	−									
4. Income	0.01	−0.02	−0.03	−								
5. Education	0.02	0.16**	0.06	0.04	−							
6. Family psychiatric	−0.04	0.14**	−0.01	0.02	0.09	−						
7. Personal psychiatric	0.01	0.14**	−0.02	−0.04	0.09	0.45**	−					
8. Close friend drinks	−0.07	−0.10*	0.09	0.10*	0.05	0.09	0.08	−				
9. Alcohol use	−0.07	0.06	0.14**	−0.01	0.16**	0.08	0.06	0.36**	−			
10. Depression score	−0.02	−0.05	0.10*	−0.20**	−0.02	0.06	0.18**	−0.01	−0.01	−		
11. Anxiety score	−0.20**	−0.11*	0.04	0.06	−0.01	0.20**	0.26**	0.18**	0.08	0.53**	−	
12. Total distress score	−0.14**	−0.09	−0.02	−0.13**	−0.01	0.16**	0.26**	0.11*	0.05	0.83**	0.91**	−

Coding structure for categorical variables: Gender (0 = female, 1 = male,); Employment (0 = unemployed, 1 = employed); Household Income (0 = less than 25 k USD, 1 = 25 k to 49 k USD, 2 = 50 k to 74 k USD, 3 = 75 k to 100 k USD, 4 = more than 100 k USD); Education (0 = some college, 1 = bachelor’s degree or higher); Family and Personal psychiatric history (0 = no, 1 = yes); Friends who drink (0 = no, 1 = yes); Alcohol user status (0 = non-drinker, 1 = drinker). Key to statistics: *p < 0.05, **p < 0.01, *t* tests (two-tailed).

### 3.2. Women who consume alcohol report more distress

**Figure 1:**
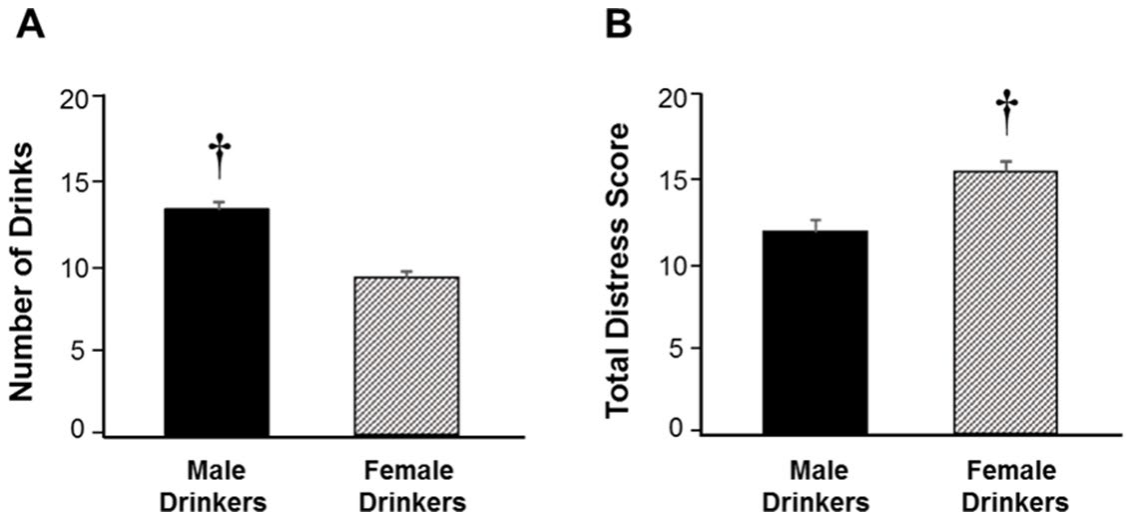
Drinking amounts and distress scores between men and women. **(A)** The figure compares average number of drinks consumed in the past 30 days between men women. Data are expressed as means ± SEM. Key to statistics: the dagger (†) denotes a significant difference between gender, with men consuming more drinks than women (*p* < 0.05). **(B)** The figure compares distress scores reported by men and women who consume alcohol. Data are expressed as means ± SEM. Key to statistics: the dagger (†) denotes a significant difference between gender with women experiencing more distress relative to men (*p* < 0.05).

**Figure 2:**
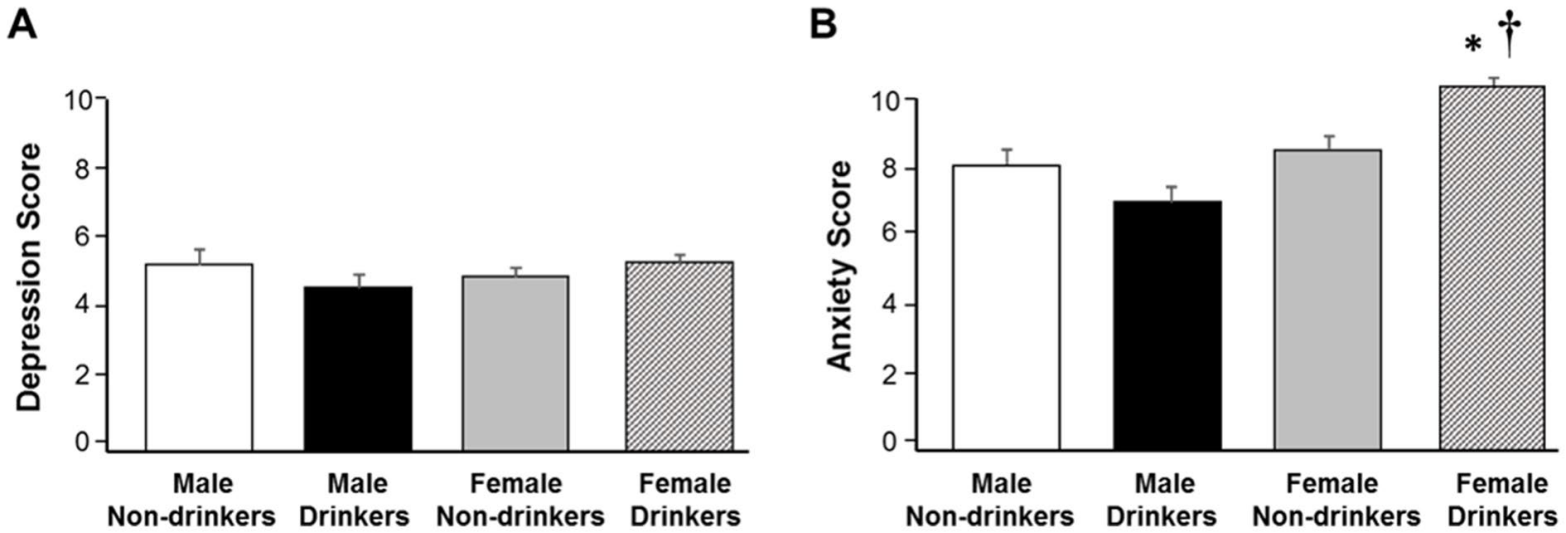
Gender differences in anxiety between drinkers and non-drinks. **(A)** The figure illustrates depression scores for men and women. Data are expressed as means ± SEM. No significant differences were observed. **(B)** The figure illustrates anxiety scores for men and women. Data are expressed as means ± SEM. Key to statistics: the dagger (†) denotes a significant difference between gender, with women who consume alcohol reporting more anxiety relative to men who also consume alcohol (*p* < 0.05). The asterisks (*) denote a significant difference between women who consume alcohol and women who do not consume alcohol (*p* < 0.05).

Figure 1A illustrates the average number of drinks consumed by men and women. Of respondents who reported alcohol use during the past 30 days, men consumed a higher number of drinks (*M* = 13.0, *SD* = 19.3) compared to women (*M* = 9.2, *SD* = 10.9), *t*(265) = 2.01, *p* < 0.05. However, the average distress scores for men and women who reported consuming alcohol differed (see Figure 1B). Specifically, significant gender differences were detected with women reporting greater cumulative distress (*M* = 15.2, SD = 6.8) compared to men (*M* = 11.9, SD = 6.3), *t*(266) = 3.49, *p* < 0.01. Figure 2A depicts depression scores separated by gender and alcohol use, however, significant differences were not detected *F*(3, 444) = 0.41, *p* = 0.74. However, there were significant differences in anxiety scores between men and women who drink *F*(3, 444) = 9.29, *p* < 0.001, with women who use alcohol reporting more anxiety relative to men (*p* < 0.001; see Figure 2B). The figure also shows that women who drink report more anxiety compared to women who do not drink alcohol (*p* < 0.01).

### 3.3. Gender differences in anxiety and alcohol consumption

Table 3 shows coefficients from a logistic regression exploring alcohol use from gender, depression scores, and anxiety scores. Gender, depression, and anxiety scores entered in Step 1 did not significantly account for variability in alcohol use, *χ*^2^(3) = 6.15, *p* = 0.10, Nagelkerke *R*^2^ = 0.02. Interactions between gender and depression scores, as well as gender and anxiety scores entered in Step 2 significantly accounted for alcohol use, Δ*χ*^2^(2) = 6.15, *p* = 0.046. Anxiety was significantly related to alcohol use for women *b* = 0.09 (*OR* = 1.10), *p* = 0.003, whereby a one-point increase in anxiety was associated with a 1.10 times greater likelihood of alcohol consumption.

**Table 3:** Logistic regression evaluating the interactions between gender, depression, anxiety scores and drinking status.

	*b*	SE_b_	Wald	OR (95% CI)	Nagelkerke *R*^2^	Δ*χ*^2^
Step 1					0.018	
Gender	−0.18	0.22	0.67	0.84 (0.55, 1.28)		
Depression scores	−0.04	0.04	1.48	0.96 (0.89, 1.03)		
Anxiety scores	0.06	0.03	4.38	**1.06** (1.04, 1.12)*		
Step 2					0.037*	6.15*
Gender	−0.31	0.22	1.93	0.73 (0.47, 1.14)		
Depression scores	−0.05	0.04	1.56	0.95 (0.87, 1.03)		
Anxiety scores	0.09	0.03	8.62	**1.10** (1.03, 1.17)**		
Gender × Depression scores	0.05	0.08	0.39	1.05 (0.90, 1.22)		
Gender × Anxiety scores	−0.15	0.06	5.41	**0.86** (0.76, 0.98)*		

Coding structure for the categorical variables of gender (0 = female, 1 = male). Coding structure for the categorical variables drinking status (0 = non-drinker, 1 = drinker). Depression and anxiety scores reflect continuous variables. Anxiety scores significantly associating with alcohol use for women. Values in parentheses denote 95% CI. Key to statistics: *p < 0.05, **p < 0.01.Bold reflect statistically significant values.

Table 4 shows coefficients from an OLS regression examining the number of alcoholic beverages consumed by participants from gender, depression scores, and anxiety scores. The final model accounted for a significant amount of variance in the total number of drinks consumed, *F*(5, 261) = 2.93, *p* = 0.014, *R*^2^ = 0.053. The interaction terms accounted for a significant amount of variance explained in the overall number of drinks consumed, Δ*F*(2, 261) = 4.44, *p* = 0.013, Δ*R*^2^ = 0.032. Interestingly, the relationship between number of drinks and anxiety score was stronger for men, *b* = 1.30 (*β* = 0.21), *p* = 0.01, indicating that among male participants who drank alcohol, the total number of drinks consumed was higher as their overall anxiety scores increased.

**Table 4:** Ordinary least squares regression evaluating interactions between gender, depression, anxiety scores and the number of drinks consumed by men and women.

	*b*	SE_b_	*t*	*β*	*R^2^*	Δ*R^2^*
Step 1					0.021	
Gender	4.00	2.00	2.00*	0.13		
Depression Scores	0.29	0.30	0.98	0.07		
Anxiety Scores	0.03	0.24	0.12	0.01		
Step 2					0.053*	0.032*
Gender	5.37	2.03	2.64**	0.17		
Depression Scores	0.29	0.34	0.85	0.07		
Anxiety Scores	−0.30	0.28	−1.10	−0.10		
Gender × Depression Scores	0.15	0.68	0.22	0.02		
Gender × Anxiety Scores	1.30	0.54	2.42*	0.21		

Coding structure for the categorical variables of gender (0 = female, 1 = male). Depression and anxiety scores reflect continuous variables. The number of alcoholic drinks consumed by participants also reflects a continuous variable. Anxiety scores were significantly more associated with the total number of drinks consumed by men. Key to statistics: *p < 0.05, **p < 0.01.Bold reflect statistically significant values.

Our approach considering the HADS cut-off point for clinical casessness revealed that of 268 participants who reported alcohol use, 17 men and 94 women met a threshold for anxiety problems. In comparison, only 2 men and 11 women who reported alcohol use met a threshold for depression problems. Table 5 shows coefficients from an OLS regression examining the number of alcoholic beverages consumed from gender and clinical cut-off thresholds. The final model accounted for a significant amount of variance, *F*(5, 261) = 4.43, *p* < 0.001, *R*^2^ = 0.078. Interaction terms accounted for a significant amount of variance explained in the number of drinks consumed, Δ*F*(2, 261) = 8.93, *p* < 0.001, Δ*R*^2^ = 0.063. The relationship between number of drinks and depression was weaker for men, *b* = −23.54 (*β* = −0.15), *p* = 0.031. In contrast, the relationship between number of drinks and anxiety was stronger for men, *b* = 17.78 (*β* = 0.32), *p* < 0.001, signifying a greater number of drinks consumed by men who reached a clinical threshold for anxiety problems.

**Table 5:** Ordinary least squares regression evaluating interactions between gender, depression caseness, anxiety caseness, and number of drinks consumed.

	*b*	SEb	*t*	*β*	*R*^2^	Δ*R*^2^
Step 1					0.015	
Gender	3.87	1.95	1.99*	0.12		
Caseness for depression	−0.41	4.00	−0.10	−0.01		
Caseness for anxiety	0.26	1.78	0.15	0.01		
Step 2					0.078*	0.063*
Gender	−0.50	2.25	−0.22	−0.02		
Caseness depression	2.87	4.22	0.68	0.05		
Caseness anxiety	−3.19	1.94	−1.65	−0.12		
Gender × Caseness for depression	−23.54	10.85	−2.17*	−0.15		
Gender × Caseness for anxiety	17.78	4.34	4.10**	0.32		

Coding structure for the categorical variables of gender (0 = female, 1 = male). Coding structure for the categorical variables reflecting potential clinical caseness for depression (0 = HADS-D scores of 10 or less, 1 = HADS-D scores of 11 or more) and anxiety (0 = HADS-A scores of 10 or less, 1 = HADS-A scores of 11 or more). The number of alcoholic drinks consumed by participants reflects a continuous variable. Anxiety scores were significantly more associated with the total number of drinks consumed by men meeting anxious symptomology. Key to statistics: *p < 0.05, **p < 0.01.Bold reflect statistically significant values.

## 4. Discussion

Previous research findings have revealed gender differences in substance abuse vulnerabilities. For example, men typically consume larger amounts of drugs, while women report greater mental health disturbances following lower rates of drug intake (Becker et al., 2017). In agreement, this study also reports that men consume more drinks compared to women; however, women who consume alcohol experience more anxiety compared to men. These observations suggest that for women, emotional dysregulation might be a key feature associated with alcohol use. In contrast, a unique relation between anxiety and drinking was observed for men, such that the total number of drinks consumed increased as men reported worsening anxiety symptoms.

Prior motivational theories exploring why people consume alcohol noted subjective emotions such as expectations of enhanced mood and reductions in negative affect as prominent drivers for drinking (Cox and Klinger, 1988). Contemporary theoretical frameworks of addiction similarly suggest that substance use vulnerability may be driven, in part, by cyclical processes of negative reinforcement produced by destabilized reward and stress systems (Koob and Schulkin, 2019). When considering these perspectives, key gender differences emerge with anxiety being a significant correlate of alcohol misuse among females (Guinle and Sinha, 2020). Indeed, the relationship between drinking initiation and anxiety is stronger for adolescent girls compared to adolescent boys (Johannessen et al., 2017). In college, anxiety coping motives are associated with drinking for women (Buckner et al., 2023), while those engaging in binge drinking report greater anxiety (Adan et al., 2016) and stress (Perrotte et al., 2018). Internalizing anxieties or experiencing traumatic events also increases the risk of developing alcohol abuse among women (Foster et al., 2015; Verplaetse et al., 2018). Women receiving inpatient clinical treatment for alcohol use disorder also report higher anxiety levels following admission and discharge compared to men (Oliva et al., 2018). Taken together with the current observations, the notion that anxiety is significantly associated with alcohol consumption among women is further supported. From a theoretical perspective, distress symptoms may influence whether or not women engage in drinking as an avoidant coping strategy against unpleasant emotion. However, given innate sex differences in absorption, distribution, and enzymatic metabolism of alcohol (Baraona et al., 2001), this approach might result in negative health outcomes, worsening of symptoms, and subsequent alcohol misuse. Further investigations are thus necessary to fully clarify the linkage between anxiety and alcohol use for women.

Anxiety is also a prominent feature associated with drinking for men. For example, men might consume alcohol to manage social anxieties (Xu et al., 2012) and, among undergraduates, men who display comorbid anxiety and depression experience more alcohol-related problems (Austin and Villarosa-Hurlocker, 2021). The latter findings align with prior observations showing a stronger association between distress and consumption for college men (Markman Geisner et al., 2004). In agreement, and perhaps one of the most revealing findings in our study, is the observation that men consumed more drinks as their anxiety worsened and reached thresholds for clinical caseness. Thus, it may be postulated that men who already consume alcohol might further drink as a maladaptive coping mechanism to ameliorate their clinical anxiety. However, because men typically drink more than women, this might produce frequent binge drinking episodes and problematic behaviors. Indeed, among college students, men who experience symptoms of anxiety represent an at-risk group characterized by heightened drug use probabilities (Martin et al., 2021) and alcohol-related troubles (Austin and Villarosa-Hurlocker, 2021).

While the present findings are consistent with previous research, our observations regarding depressive symptoms and alcohol use remain elusive. For instance, there were no gender differences between those who consumed alcohol and depression scores. In addition, the small number of men who reached a clinical threshold for depression reported drinking less. In contrast, data from college settings does indicate a co-occurrence between alcohol use and depression (Pedrelli et al., 2016; Kenney et al., 2017; Villarosa et al., 2018). In addition, epidemiological data suggests that a history of drinking can lead to depression (McHugh and Weiss, 2019). Given this comorbidly and directionally, perhaps studying a larger sample with depressive symptoms, or a clinical population, might have provided better insight. Nonetheless, evidence does show that college men with depressive symptoms engage less in drug use (Martin et al., 2021), raising the possibility that different substances prompt unique phenotypical reactivities in combination with depression.

We also found some sociodemographic characteristics linked with alcohol consumption. Drinking alcohol is a prevalent behavior among US college students (Lorant et al., 2013; White and Hingson, 2013). Not surprisingly, our study also reports that the number of drinks consumed in the past 30 days is significantly increased for participants with some college experience relative to those with less college experience. We also noted a moderate correlation between drinking and having a close friend who consumes alcohol. This is consistent with evidence indicating associations between alcohol use and peer influences among undergraduate students (Walther et al., 2017).

### 4.1. Limitations and future direction

Although our study further provides insight to the relationship between distress symptoms, gender, and alcohol consumption, there are limitations to consider. First, this study was conducted using self-report measurements. A common disadvantage associated with survey assessments is their tendency toward generating biased responses centered on socially acceptable answers or preferred self-appraisals (Althubaiti, 2016). Second, because of the cross-sectional approach used in this study, determining causal relationships is improbable. Third, a comprehensive diagnosis for psychopathology or alcohol use disorder was not directly evaluated and thus our results may not generalize to clinical populations with existing psychiatric conditions. Fourth, different types of beverages consumed by participants were not considered, hence, discussions related to alcoholic content by volume are limited. Lastly, other factors like sensation seeking, behavioral disinhibitions (Adan et al., 2016; Obst et al., 2021), and those affecting mental health such as circadian phenotypes (Díaz-Morales et al., 2017), may also influence drinking behavior. Indeed, it is thought that merely drinking to cope with negative emotions may not fully explain alcohol consumption, but rather independent variables combine to develop alcohol-related harm (Boyd et al., 2022; Shuai et al., 2022). Therefore, future research may consider evaluating alcohol use from a multifaceted approach.

### 4.2. Conclusion

The present study revealed that for college women, the mere presence of anxiety symptoms associates with a likelihood of alcohol use. Given that women experience distinct alcohol-related social (Dardis et al., 2021) and health (Erol and Karpyak, 2015) consequences, college systems might consider screening for emotional dysregulation as a preventative method to lower drinking outcomes. Another strength of our report is the observation that among college men, heightened symptoms of anxiety associate with greater alcohol consumption. Thus, educational measures might also convey emotional management skills to reduce drinking disparities within susceptible populations. These prioritized intervention approaches might also benefit individuals with other emotional instabilities, such as impulsivity and co-dependency, thought to mediate problems observed in college like excessive internet use (Diotaiuti et al., 2022). Taken together, our report highlights the necessity to investigate gender-specific associations between distress symptoms and substance use. Elucidating the multifaceted aspect of alcohol use can provide reformative knowledge about drinking, and guide the development of gender-specific strategies to reduce alcohol use vulnerabilities.

## Data availability statement

Raw data supporting the conclusions of this article are available by the authors upon request.

## Ethics statement

The studies involving human participants were reviewed and approved by SDCCD Institutional Review Board approval (IRB# 2019010). The patients/participants provided their written informed consent to participate in this study.

## Author contributions

OT developed the study design and manuscript concept. MG, NA, OT, and LV performed the data collection. JE and OT conducted the statistical analyzes and interpretations. OT, NA, and GT conducted the manuscript revision and editing. All authors reviewed and approved the final version of this manuscript.

## Conflict of interest

The authors declare that the research was conducted in the absence of any commercial or financial relationships that could be construed as a potential conflict of interest.

## Publisher’s note

All claims expressed in this article are solely those of the authors and do not necessarily represent those of their affiliated organizations, or those of the publisher, the editors and the reviewers. Any product that may be evaluated in this article, or claim that may be made by its manufacturer, is not guaranteed or endorsed by the publisher.
